# Impaired consciousness at stroke onset in large hemisphere infarction: incidence, risk factors and outcome

**DOI:** 10.1038/s41598-020-70172-1

**Published:** 2020-08-05

**Authors:** Jie Li, Ping Zhang, Simiao Wu, Ruozhen Yuan, Junfeng Liu, Wendan Tao, Deren Wang, Ming Liu

**Affiliations:** 1 Department of Neurology, Center of Cerebrovascular Diseases, West China Hospital, Sichuan University, No. 37 GuoXue Xiang, Chengdu, 610041 Sichuan Province People’s Republic of China; 2 Department of Neurology, People’s Hospital of Deyang City, No. 173, North Taishan Road, Deyang, 618000 Sichuan Province People’s Republic of China

**Keywords:** Neuroscience, Diseases of the nervous system, Stroke

## Abstract

Impaired consciousness (IC) at stroke onset in large hemispheric infarctions (LHI) patients is common in clinical practice. However, little is known about the incidence and risk factors of IC at stroke onset in LHI. Besides, stroke-related complications and clinical outcomes in relation to the development of IC has not been systematically examined. Data of 256 consecutive patients with LHI were collected. IC at stroke onset was retrospectively collected from the initial emergency department and/or admission records. Of the 256 LHI patients enrolled, 93 (36.3%) had IC at stroke onset. LHI patients with IC at stroke onset were older (median age 66 vs. 61, *p* = 0.041), had shorter prehospital delay (24 vs. 26 h, *p* < 0.001and higher baseline National Institutes of Health Stroke Scale (NIHSS) score (19 vs. 12, *p* < 0.001). Independent risk factors of IC at stroke onset were high NIHSS score (odds ratio, OR 1.17, 95% confidence interval [CI] 1.12 to 1.23) and atrial fibrillation (OR 1.93, 95% CI 1.07 to 3.47). Dyslipidemia appeared to protect against IC at stroke onset (adjusted OR 0.416, 95% CI 0.175 to 0.988). IC at stroke onset was associated with higher frequency of stroke-related complications (90.32% vs. 67.48%, *p* < 0.001), especially brain edema (45.16% vs. 23.31%, *p* < 0.001) and pneumonia (63.44% vs. 47.82%, *p* = 0.019). The IC group had higher rates of in-hospital death (23.66% vs. 11.66%, *p* = 0.012), 3-month mortality (49.46% vs. 24.87%, *p* = 0.002), and 3-month unfavorable outcome (64.51% vs. 49.07%, *p* = 0.017). However, after adjusting for age, baseline NIHSS score and other confounders, IC at stroke onset was not an independent predictor of in-hospital death (adjusted OR 0.56, 95% CI 0.22 to 1.47), 3-month mortality (adjusted OR 0.54, 95% CI 0.25 to 1.14) and 3-month unfavorable outcome (adjusted OR 0.64, 95% CI 0.31 to 1.33) in LHI patients (all *p* > 0.05). Our results suggested that IC occur in 1 out of every 3 LHI patients at stroke onset and was associated with initial stroke severity and atrial fibrillation. LHI patients with IC at stroke onset more frequently had stroke-related complications, 3-month mortality and unfavorable outcome, whereas IC was not an independent predictor of poor outcomes.

## Introduction

Consciousness is defined as the presence of a wakeful arousal state and the awareness to internal and external events^1^. Both aspects of consciousness depend on the integrity of the ascending reticular activating system (ARAS) in the upper brainstem tegmentum (reticular formation) and central thalamus, which promoted widespread cortical activation^2^. Coma and other states of impaired consciousness (IC) are frequent in stroke patients. Stroke is one of the three most common causes of IC in emergency rooms, together with trauma and hypoglycemia^3^.In an observational study of non-traumatic coma patients, cerebrovascular disease accounted for 50% of the cases^4^. Data from an emergency room-based study of stroke coma suggested that the leading cause of stroke coma was intracerebral hemorrhage (72%), followed by cerebral infarction (23%) and subarachnoid hemorrhage (5%)^5^.

IC is common in the early stage of ischemic stroke. In one of our published studies, 35% of ischemic stroke patients admitted to hospital within 24 h from onset experienced IC on admission, and among those patients, 62.3% of cases were finally diagnosed as large hemispheric infarction (LHI), which supported that early IC indicates the presence of large hemispheric lesions^6^. It is known that sudden impairment of consciousness can be the predominant clinical manifestation in brainstem stroke, particularly in basilar artery occlusion. It is reported that 33% of basilar embolism patients had IC at stroke onset^7^. Despite the lack of research, IC at stroke onset in LHI patients is also common in clinical practice. However, little is known about the incidence and risk factors of IC at stroke onset in LHI.

LHI, which usually caused by occlusion of the internal carotid artery or proximal middle cerebral artery (MCA), constitutes up to 10% of supratentorial ischemic strokes^8^. It is a widely accepted devastating condition with a high mortality rate of approximately 80% in two intensive care-based series^9,10^. LHI is commonly associated with varying degrees of brain swelling, with subsequent raised intracranial pressure, midline shift and brain herniation, giving rise to the term malignant MCA infarction (mMCAI)^11^. Within 24–48 h of onset, there is usually a progressive clouding of consciousness owing to the brain edema, which deteriorates to coma and brain death within 2 to 5 days in most mMCAI patients^12^. Up to now, no pharmacological strategies have been proven effective by clinical trials^13^. Decompressive hemicraniectomy (DHC) conducted within 48 h after symptom onset has been proposed as a treatment option for LHI patients with malignant course^14^. Since DHC is an aggressive therapeutic approach, the identification of those patients most likely to have a poor outcome would help optimize patient selection for this aggressive treatment.

Although it is widely accepted that brain edema and mass effect are often associated with IC in LHI patients, the clinical application of IC as a predictor of outcome after major stroke remains controversial^15–17^. Meanwhile, previous studies of IC after stroke usually assessed consciousness states at the time of hospital admission or during hospitalization. For these reasons, the present study aimed to identify the incidence and risk factors associated with IC at stroke onset in LHI patients and to explore their influence on stroke-related complications and outcomes.

## Methods

### Study design and subjects

From October 2011 to September 2014, we prospectively and consecutively enrolled acute ischemic stroke patients admitted to the Department of Neurology, People’s Hospital of Deyang City. LHI was defined as infarction involving more than 50% of MCA region in computed tomography (CT) and/or magnetic resonance imaging (MRI), no matter the involvement of the adjacent territories^18^. All patients completed brain CT before initial treatment. A second CT or MRI was performed within the first 7 days of hospitalization. Other CT scans were performed if patients suffered neurological deterioration, to identify brain edema or hemorrhagic transformation. Patients with incomplete hospital records or missing imaging that would prevent complete data collection were excluded. We also excluded cases with a premorbid modified Rankin scale (mRS, a scale of 0 to 6, with 0 indicating no symptoms and 6 indicating death) score of more than 2 and lived dependently^19^.

The Ethics Committee of the people's hospital of Deyang city approved the study protocol (Approval No. 2011-04-134). We obtained informed consent from all patients or their legal representative if the patient lost the capacity to give informed consent before they were enrolled. All methods in the current study were conducted in accordance with relevant guidelines and regulation.

### Data collection and outcome

A standardized structured form was used to collect the following data: patient demographics, prehospital delay, baseline blood pressure and serum glucose, initial stroke severity (assessed by baseline NIHSS score), vascular risk factors, diagnostic tests, neurological imaging, in-hospital treatment, and stroke-related complications during hospitalization. Detailed methods for data collection have been described in our previous study^20^. The potential stroke etiology of LHI was categorized as large-artery atherosclerosis, cardio-embolism, the stroke of other determined etiology and stroke of undetermined etiology according to the Trial of Org 10,172 in Acute Stroke Treatment (TOAST) criteria^21^. In-hospital treatments analyzed in our study included thrombolysis, mechanical ventilation, osmotic agents (such as mannitol), and DHC. Stroke-related complications, including both neurological and medical complications during hospitalization^22^, were reviewed from hospital records when the patient was discharged by data collectors who were not aware of the study, which has been elaborated upon in our previous study^23^.

Consciousness states at stroke onset were retrospectively collected from the initial emergency department records and/or admission records by two experienced neurologists independently. Impaired consciousness and coma were usually recorded according to Adams and Victor's Principles of Neurology (9th edition) in our clinical practice^24^, via evaluating the patient's wakefulness, verbal and motor response, and orientation to person, place and time. For LHI patients with an unknown time of symptom onset, including wake-up strokes and unwitnessed day-time strokes, consciousness states were assessed at the first noticed abnormal time. If there was any disagreement, further detailed medical history inquiry of the witness about the history of consciousness at the onset of stroke would be conducted to reach a consensus. LHI patients assessed with definitely IC at stroke onset, regardless of transitory impaired consciousness, minimally conscious state, or coma, were assigned to the IC group, while those patients assessed without definitely IC were assigned to the ‘without IC’ group.

Patients were followed up at 3-month after stroke onset by telephone interview or letter inquiries via using questionnaires. The primary outcomes were in-hospital death, 3-month mortality and the 3-month unfavorable outcome (defined as a mRS score of 4 to 6)^19^.

### Statistical analyses

Intergroup differences in categorical variables were assessed for significance using the χ^2^ tests or Fisher’s exact tests, while differences in continuous variables were assessed using the student's t-tests or Mann–Whitney U test. Univariate analysis comparing putative risk factors between LHI patients with and without IC at stroke onset was performed. Multivariate logistic regression analyses via the Forward LR procedure was used to identify risk factors of IC at stroke onset. In addition, we performed stratified analyses to explore potential interactions between identified risk factors. The significance of interaction was tested by the Log likelihood ratio test. Univariate analysis was performed to test variables that may affect the outcome. The odds ratios (ORs) for variables associated with 3-month mortality and unfavorable outcomes were identified via multivariable logistic regression analyses by using the forced entry method adjusted for variables with *p* < 0.1 in univariate analyses. 3-month survival was estimated by the Kaplan–Meier method and a log-rank test was used for survival comparisons between patient groups. The 95% confidence intervals (CI) were calculated to describe the precision of the estimates. Two-sided *p* < 0.05 was considered to be statistically significant. All statistical analysis was performed using SPSS v21.0 (IBM, Chicago, IL, USA), the statistical software packages R (https://www.R-project.org, The R Foundation, version 3.4.3) and EmpowerStats (https://www.empowerstats.com, X&Y Solutions, Inc., Boston, MA, USA), which have been described in our previous studies^6,20^.

## Results

Between October 2011 and September 2014, 1,542 acute ischemic stroke patients were consecutively and prospectively registered. Among those patients, 256 (16.6%) cases with LHI were enrolled in the current study, comprising 123 men and 133 women aged 61.6 ± 15.3 years (median NIHSS score on admission: 14). Of the 256 LHI patients enrolled, 93 (36.3%) had IC at stroke onset, finally 14 patients regained consciousness at their arrival to our hospital; 163 (63.7%) did not have IC at stroke onset, however, 58 patients developed IC during the time between stroke onset and their arrival to our hospital.

### Characteristics of LHI patients with and without IC

The baseline characteristics of LHI patients with and without impaired consciousness at stroke onset were compared in Table 1. LHI patients with IC at stroke onset were older (median age 66 vs. 61, *p* = 0.041), had shorter prehospital delay (24 vs. 26 h, *p* < 0.001) and higher median NIHSS score (19 vs. 12, *p* < 0.001) on admission. LHI patients with IC at stroke onset also showed higher rates of some stroke risk factors [atrial fibrillation (55.91% vs. 36.20%, *p* = 0.002) and history of previous ischemic stroke (16.13% vs. 7.36%, *p* = 0.035)], but a lower rate of hyperlipidemia (10.75% vs. 22.70%, *p* = 0.018). Compared with LHI patients without IC at stroke onset, the IC group had a higher rate of impaired consciousness (84.95% vs. 35.58%, *p* < 0.001) and coma (77.42% vs. 47.85%, *p* < 0.001) on admission. Meanwhile, the IC group had a higher rate of dominant hemisphere involvement (60.22% vs. 43.56%, *p* = 0.013).

**Table 1:** Characteristics and in-hospital treatment of LHI patients with and without impaired consciousness at stroke onset.

	IC (n = 93)	Without IC (n = 163)	*P* value
Age (years), median(range)	66 (17–99)	61 (15–93)	0.041^†^
Female, n (%)	51 (54.84)	82 (50.31)	0.517^‡^
Rural population, n (%)	24 (25.81)	61 (37.42)	0.073^‡^
Time from onset(hours), median(range)	24 (1–504)	26 (3–720)	< 0.001^†^
NIHSS score on admission, median(range)	19 (5–33)	12 (4–31)	< 0.001^†^
IC on admission	79 (84.95)	58 (35.58)	< 0.001^‡^
Coma on admission	48 (51.61)	18 (11.04)	< 0.001^‡^
SBP on admission (mm Hg)	143.55 ± 31.10	139.17 ± 22.73	0.236*
DBP on admission (mm Hg)	85.08 ± 17.53	83.41 ± 14.95	0.422*
Serum glucose on admission(mmol/L)	8.07 ± 3.83	7.59 ± 3.00	0.272*
**Risk factors, n (%)**			
Hypertension	44 (47.31)	85 (52.15)	0.457^‡^
Diabetes mellitus	19 (20.43)	34 (20.86)	0.935^‡^
Dyslipidemia	10 (10.75)	37 (22.70)	0.018^‡^
Coronary heart disease	11 (11.83)	19 (11.66)	0.967^‡^
Atrial fibrillation	52 (55.91)	59 (36.20)	0.002^‡^
Rheumatic heart disease	26 (27.96)	32 (19.63)	0.126^‡^
Current smoking	17 (18.28)	41 (25.15)	0.219^‡^
Alcohol consumption	13 (13.98)	29 (17.79)	0.486^‡^
Previous IS	15 (16.13)	12 (7.36)	0.035^‡^
Previous TIA	1 (1.08)	6 (3.68)	0.406^‡^
Previous ICH	4 (4.30)	1 (0.61)	0.114^‡^
Stroke in dominant hemisphere, n (%)	56 (60.22)	71 (43.56)	0.013^‡^
**TOAST classification, n (%)**			0.004^‡^
Large-artery atherosclerosis	13 (13.98)	46 (28.22)	
Cardio-embolism	55 (59.14)	68 (41.72)	
Other determined etiology	8 (8.60)	6 (3.68)	
Undetermined etiology	17 (18.28)	43 (26.38)	
**In-hospital Treatments, n (%)**			
Thrombolysis	2 (2.15)	5 (3.07)	0.973^‡^
Decompressive surgery	14 (15.05)	10 (6.13)	0.019^‡^
Mechanical ventilation	17 (18.28)	13 (7.98)	0.014^‡^
osmotic agents	86 (92.47)	119 (73.01)	< 0.001^‡^
Days of hospitalization, Mean ± SD	13.53 ± 12.79	11.32 ± 9.30	0.147*

IC, impaired consciousness at stroke onset; SBP, systolic blood pressure; DBP, diastolic blood pressure; NIHSS, National Institutes of Health Stroke Scale; IS, ischemic stroke; ICH, intracerebral hemorrhage; TIA, transient ischemic stroke; SD, standard deviation.

*Student t test.

^†^Mann–Whitney U test.

^‡^χ2 test.

According to the TOAST criteria, the stroke etiology of LHI was significantly different between the two groups (*p* = 0.004). The most common stroke etiology in the IC group was cardio-embolism (59.14%), followed by the stroke of undetermined etiology (18.28%), large-artery atherosclerosis (13.98%), and stroke of other determined etiology (8.60%).

For the acute phase treatment of LHI, patients with IC at stroke onset were more frequently to use osmotic agents (92.47% vs. 73.01%, *p* < 0.001) and to receive mechanical ventilation (18.28% vs. 7.98%, *p* = 0.014) and DHC (15.05% vs. 6.13%, *p* = 0.019).

### Risk factors of IC at stroke onset

Multivariable logistic regression identified two independent risk factors of IC at stroke onset in LHI patients (Table 2): baseline NIHSS score (adjusted OR 1.174, 95% CI 1.117 to 1.234) and atrial fibrillation (adjusted OR 1.930, 95% CI 1.072 to 3.475). Conversely, dyslipidemia appeared to protect against IC at stroke onset (adjusted OR 0.416, 95% CI 0.175 to 0.988). Age, history of previous ischemic stroke, stroke in dominant hemisphere and TOAST classification were not independently associated with IC at stroke onset in LHI patients. After adjustment for potential confounding variables, there was no evidence for an interaction between atrial fibrillation, dyslipidemia, and baseline NIHSS score on the incidence of IC at stroke onset. We found that the association between atrial fibrillation and IC did not change by baseline NIHSS score and dyslipidemia, meanwhile, the association between dyslipidemia and IC did not change by baseline NIHSS score and atrial fibrillation (Supplemental Tables 1 and 2, all *p* for interaction > 0.05).

**Table 2:** Factors associated with impaired consciousness at stroke onset in LHI patients.

Variables	Univariate analysis	Multivariate analysis*
Age	1.014 (0.997–1.032)	
Baseline NIHSS score	1.178 (1.122–1.238)	1.174 (1.117–1.234)
Dyslipidemia	0.410 (0.194–0.870)	0.416 (0.175–0.988)
Atrial fibrillation	2.236 (1.330–3.758)	1.930 (1.072–3.475)
Previous IS	2.420 (1.080–5.422)	
Stroke in dominant hemisphere	1.961 (1.168–3.292)	
**TOAST classification**		
Large-artery atherosclerosis	Reference	
Cardio-embolism	2.862 (1.406–5.826)	
Other determined etiology	4.718 (1.387–16.053)	
Undetermined etiology	1.399 (0.608–3.219)	

NIHSS, National Institutes of Health Stroke Scale; IS, ischemic stroke.

Variables that had a potential association with impaired consciousness at stroke onset in univariate analysis (*P* < 0.1) were listed and included in the multivariate logistic regression using the Forward LR method. Hosmer and Lemeshow Test (*P* = 0.185).

*Adjusted odds ratios (OR) with *P*  < 0.05 in the multivariate logistic regression analysis. Figures in parentheses are 95% confidence intervals (CI).

### Stroke-related complications

Table 3 lists stroke-related complications during hospitalization of LHI patients with and without IC at stroke onset. Overall, LHI patients with IC at stroke onset more frequently had stroke-related complications (90.32% vs. 67.48%, *p* < 0.001). The IC group had a significant higher rate of malignant brain edema (45.16% vs. 23.31%, *p* < 0.001), hemorrhagic transformation (36.56% vs. 22.70%, *p* = 0.020) and seizures/epilepsy (13.98% vs. 3.68%, *p* = 0.009). However, there was no significant difference in the events rate of central hyperthermia and recurrent stroke between groups (all *p* > 0.05). With respect to the medical complications, pneumonia was the most common medical complication in both groups (63.44% in patients with IC at stroke onset and 47.82% in those without IC at stroke onset). Moreover, the IC group had significantly higher event rates of pneumonia, electrolyte disturbance and urinary incontinence (all *p* < 0.05).

**Table 3:** Stroke-related complication during hospitalization of LHI patients with and without impaired consciousness at stroke onset.

	IC (n = 93)	Without IC (n = 163)	*P* value
Complications, n (%)	84 (90.32)	110 (67.48)	** < **0.001
**Neurological complications, n (%)**			
Brain edema	42 (45.16)	38 (23.31)	** < **0.001
Hemorrhagic transformation	34 (36.56)	37 (22.70)	0.020
Seizures/epilepsy	12 (13.98)	6 (3.68)	0.009
Central hyperthermia	5 (5.38)	6 (3.68)	0.747
Recurrent stroke	0 (0)	3 (1.84)	0.556*
**Medical complications, n (%)**			
Pneumonia	59 (63.44)	78 (47.82)	0.019
Urinary tract infection	6 (6.45)	13 (7.98)	0.806
Gastrointestinal bleeding	10 (10.75)	20 (12.27)	0.841
Electrolyte disturbance	38 (40.86)	41 (25.15)	0.011
Acute renal failure	9 (9.68)	9 (5.42)	0.309
Urinary incontinence	27 (29.03)	20 (12.27)	** < **0.001
Bedsore	7 (7.53)	6 (3.68)	0.293
Deep venous thrombosis	6 (6.45)	3 (1.84)	0.116
Falls	1 (1.08)	2 (1.23)	1.000*

*Fisher exact test.

### Outcomes

**Figure 1:**
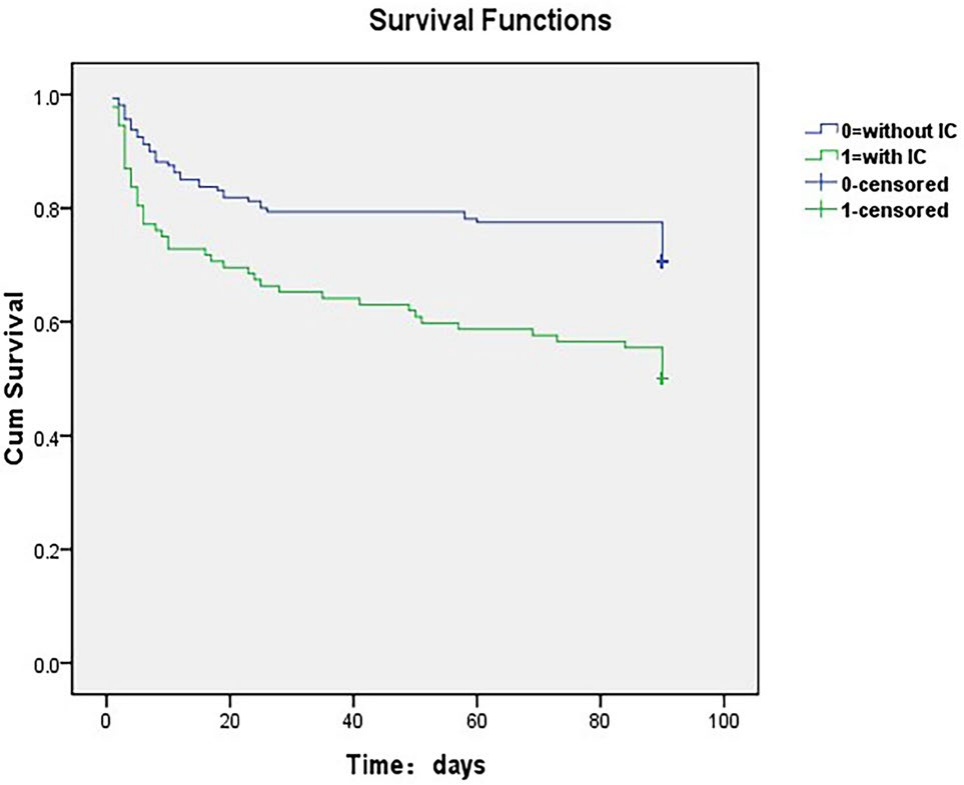
3-month survival curves for LHI patients with or without impaired consciousness at stroke onset.

Outcomes of LHI patients with IC at stroke onset or not were shown in Table 4. LHI patients with IC at stroke onset showed significantly higher rates of in-hospital death (23.66% vs. 11.66%, *p* = 0.012). At 3 months, 1.2% (3/256) patients were lost to follow-up, among which one patient was in the IC group and two patients were in the without IC group, respectively (1.08% vs. 1.23%, *p* = 0.914). Among the entire cohort, 94 (36.7%) patients died at 90 days and 140 (55.3%) had unfavorable outcomes. LHI patients with IC at stroke onset showed higher rates of 3-month mortality (49.46% vs. 24.87%, *p* = 0.002) and 3-month unfavorable outcome (64.51% vs. 49.07%, *p* = 0.017) than those without IC at stroke onset. The 3-month survival rates of LHI patients with IC at stroke onset were significantly lower than those without (*p* = 0.001, log-rank test; Fig. 1). Comparing the outcome of IC group in dominant versus non-dominant hemispheres, the IC group involving non-dominant hemispheres had a higher rate of 3-month unfavorable outcome (75.68% vs. 57.14%, *p* = 0.043, Supplemental Table 3). After adjusting for age, baseline NIHSS score, stroke risk factors, in-hospital treatment and stroke-related complications which had potential confounding effects on the clinical outcome, IC at stroke onset was not independent predictor of in-hospital death (adjusted OR 0.56, 95% CI 0.22 to 1.47), 3-month mortality (adjusted OR 0.54, 95% CI 0.25 to 1.14) and 3-month unfavorable outcome(adjusted OR 0.64, 95% CI 0.31 to 1.33) in LHI patients (all *p* > 0.05).

**Table 4:** Outcome of LHI patients with or without impaired consciousness at stroke onset.

	IC (n = 93)	Without IC (n = 163)	OR (95%CI)	Adjusted OR (95%CI)
Death in-hospital	22 (23.66)	19 (11.66)	2.35 (1.19–4.62)	0.56 (0.22–1.47)*
3-month case-fatality	46 (49.46)	48 (24.87)	2.33 (1.37–3.97)	0.54 (0.25–1.14)*
3-month unfavorable outcome	60 (64.51)	80 (49.07)	1.90 (1.12–3.22)	0.64 (0.31–1.33)*

*Adjusted for age, baseline NIHSS score, baseline serum glucose and stroke risk factors which had potential confounding effects on the clinical outcome in univariate analysis.

## Discussion

Nowadays, there is scarce information available regarding the incidence and risk factors of IC at stroke onset in LHI, the relationship between IC at stroke onset and stroke-related complications and clinical outcomes in LHI patients has not been systematically examined. In the present study, we found that IC occurred in 1 out of every 3 LHI patients at stroke onset. Independent risk factors of IC at stroke onset were initial stroke severity assessed by baseline NIHSS score and atrial fibrillation, while dyslipidemia appeared to protect against IC at stroke onset. IC at stroke onset was associated with a higher frequency of stroke-related complications, especially brain edema and pneumonia. LHI patients with IC at stroke onset had higher rates of in-hospital death, 3-month mortality, and 3-month unfavorable outcome. However, after adjusting for age, baseline NIHSS score and other confounders, IC at stroke onset was not an independent predictor of clinical outcome in LHI patients.

Although sudden impairment of consciousness at stroke onset in LHI patients is common in clinical practice, the incidence and risk factors of IC at stroke onset in LHI patients are poorly understood. Previous studies of IC after stroke usually assessed consciousness states at the time of hospital admission or after hospitalization. Data from various stroke researches suggested that about 14.4–53.8% of large supratentorial infarction patients experienced IC on admission when admitted to hospital within 6–48 h from onset^15,17,25–28^. In a published cohort of 564 placebo-treated patients with major anterior circulation infarction, IC was present in 409 patients (72.5%) during the 24-h time period^29^. Another cohort of 208 LHI patients based on the Lausanne stroke registry reported that 55% of cases experienced IC within 24 h after admission^30^. Here we provide evidence that IC at stroke onset is common in LHI patients, occurring in more than one thirds of LHI patients in our cohort. Differences in the incidence of IC between our study and others could be easily explained by the heterogeneity in the time between stroke onset and hospital admission. Because different pre-hospital care, disease course and stroke-related complications could contribute to different consciousness states at time of admission. It is worth noting that time interval between stroke onset and hospital arrival in our study seems to be very long, the median prehospital delay is 24 versus 26 h in the two groups respectively. This might be explained by that the current study was a hospital-based study conducted in a large (Level III) hospital that covering over 4 million local residents in western China, so some patients were transferred from smaller hospitals for advanced treatment. Furthermore, more than one fourth of LHI patients came from rural areas in our study, and many patients were lack of knowledge of the importance for seeking immediate medical service after onset of stroke.

In the present study, we found that LHI patients with IC at stroke onset showed a higher rate of atrial fibrillation, and the most common stroke etiology in the IC group was cardio-embolism (59.14%). Meanwhile, multivariable logistic regression identified atrial fibrillation as an independent risk factor of IC at stroke onset in LHI patients (1.930, 95% CI 1.072 to 3.475), in addition to initial stroke severity. Stratified analyses and test of interaction showed that the association between atrial fibrillation and IC did not change by baseline NIHSS score. These results are consistent with a study comparing the early clinical signs of severe atherothrombotic and cardioembolic infarction, which suggested that IC at stroke onset was 3.2-fold more frequent in cardio-embolic infarction than in atherothrombotic infarction^31^. Besides, the European Community Stroke Project reported that the incidence of coma and confusion within a week of stroke onset was higher in patients with atrial fibrillation^32^. IC at stroke onset usually pointed to a cardiac origin of the stroke especially those caused by atrial fibrillation. This could be explained by two reasons. First, cardiac emboli are often large and therefore more likely to affect the main arteries of the cerebrovascular system and then cause more wide spread lesion, and more frequent hemorrhagic transformation^33^. Second, it has been proven that lateral displacements of the brain above the tentorium are closely related to IC. Since infarcts are larger and probably more inferiorly located in Cardio-embolic stroke, the lateral shift may be more significant and could account for the increased frequency of IC^34–35^.

It's worth noting that, dyslipidemia appeared to protect against IC at stroke onset in our study (adjusted OR 0.416, 95% CI 0.175 to 0.988). A large cohort study conducted in Danmark have reported that higher total serum cholesterol levels are associated with less severe strokes. The authors of that study offered a possible explanation that patients with atrial fibrillation (related to embolic and more severe strokes) had markedly lower cholesterol values than those patients without atrial fibrillation^36^. Interestingly, in the present study, stratified analyses and test of interaction showed that the association between dyslipidemia and IC did not change by baseline NIHSS score and atrial fibrillation. So, our result could not be explained by that dyslipidemia is more frequent in patients with atherothrombotic infarction, and thus those patients are less severe and less likely to have atrial fibrillation. The possible explanation for our finding which might be reported as "lipid-paradox" remains unclear. It might be the result of a balance between the disadvantages associated with dyslipidemia that lead to ischemic stroke and the benefits associated with dyslipidemia that protect against severe stroke. The paradox could also be explained as an artificial finding simply due to selection bias.

Stroke-related complications, both neurological and medical, are major causes of morbidity and mortality after ischemic stroke. Moreover, medical and neurological complications can hinder rehabilitation, prolong hospitalization and increase healthcare costs^37–38^. The present cohort identified that IC at stroke onset was associated with an elevated risk of stroke-related complications in LHI patients, especially brain edema and pneumonia. It has been demonstrated that brain edema is the principal cause of death and poor functional outcome in patients with LHI^11^. The predictors of malignant brain edema using clinical and radiological variables have been thoroughly investigated for the last two decades. Systematic reviews have demonstrated that younger age, higher baseline NIHSS, and infarct size were major predictors for malignant edema after stroke^39–40^. However, whether IC on admission associated with malignant brain edema or not remains controversial^10,17,25–28,41^. Our cohort associated IC at stroke onset with an elevated risk of brain edema. Although this result still requires further investigation, given the higher risk of brain edema, more frequent monitoring of consciousness and other neurological signs in LHI patients with IC at stroke onset is of great importance for their correct management.

It is demonstrated that pneumonia is the major early complication that is associated with high morbidity and mortality in stroke patients^42,43^. An observational study conducted in LHI patients has identified pneumonia as the most common stroke-related complication and an independent predictor of 3-month unfavorable outcome^23^. Data from China National Stroke Registry (CNSR) indicated that that pneumonia might be an essential risk factor for the development of several non-pneumonia medical complications after stroke^44^. However, the administration of prophylactic antibiotics has not been proven effective by clinical trials in acute stroke patients^45–46^. As a result, studies on the predictors of post-stroke pneumonia would be useful to put appropriate preventive strategies in place. In a cohort study of acute stroke patients fed by nasogastric, a decreased level of consciousness was identified to be an independent clinical feature predictive of pneumonia^47^. In our cohort, we identified LHI patients with IC at stroke onset had a significantly higher rate of pneumonia. Although early screening for dysphagia is of great importance for the prevention of pneumonia in acute stroke patients, dysphagia might not be testable in most IC patients. Nevertheless, previous studies have suggested NIHSS score a simple and reliable predictor of dysphagia since a NIHSS of 11.5 was a cutoff between transient and persistent dysphagia while 4.5 was a cutoff between no dysphagia and dysphagia^48–50^. Therefore, using NIHSS score to estimate the risk of dysphagia in IC patients could be an alternative option. In addition, after hospital admission of LHI patients with IC at stroke onset, other appropriate preventive strategies including good nursing, handwashing, and aseptic precautions should be considered to prevent pneumonia and other complications.

In the present study, we found that LHI patients with IC at stroke onset had higher rates of in-hospital death, 3-month mortality and 3-month unfavorable outcome. However, after adjusting for age, baseline NIHSS score and other confounders, IC at stroke onset was not an independent predictor of clinical outcomes in LHI patients. A cohort study of 1029 patients undergoing stroke rehabilitation has indicated that greater neurological deficit is associated with the higher frequency of stroke-related complications and neurological impairment level is the strongest predictor of the frequency of complications^51^. Meanwhile, patients with severe neurological deficits are particularly susceptible to many medical complications^22,52^. On the other hand, our previous study suggests that brain edema and pneumonia are independent predictors of 3-month unfavorable outcome in LHI patients, in addition to age and baseline NIHSS score^23^. Since baseline NIHSS score was significantly higher among LHI patients with IC at stroke onset than among patients without it in our cohort, we could reasonably explain the result of multivariate analysis that it was the higher baseline NIHSS score and increased risk of stroke-related complications especially for brain edema and pneumonia, rather than IC at stroke onset independently associated with unfavorable outcome in LHI patients. For these reasons, the fact that IC was not a risk factor for death and unfavorable outcome after correcting for these factors is not surprising. However, since IC at stroke onset is a meaningful sign of increased stroke severity and higher risk of stroke-related complications, it might be of great importance when developing treatment and management strategies in LHI patients.

The present study has several limitations, so our results should be interpreted with caution. First, IC at stroke onset in our study were retrospectively collected from the initial emergency department records and/or admission records, so we could not provide accurate information on the duration of the IC, which could be related with outcome. Second, although use of consensus of experienced neurologists via delayed medical history inquiry of the witness, retrospective collection of IC at stroke onset might still be inaccurate, as other stroke manifestations, such as aphasia, neglect and eye lid opening apraxia, might be mistaken for IC. However, just for this reason, our results could better represent the clinical characteristics and outcomes of LHI patients with IC at stroke onset judged by the general public. In the present study, comparing the outcome of IC group in dominant versus non-dominant hemispheres, the IC group involving non-dominant hemispheres had a higher rate of 3-month unfavorable outcome. As a result, IC at stroke onset might be a more meaningful sign in LHI patients involving non-dominant hemispheres, when developing treatment and management strategies. Third, it was a single tertiary hospital-based study, so the results may not represent the whole population. Some patients with severe stroke might not be hospitalized, especially those who died before hospitalization, so we could not exclude inclusion bias. Fourth, we only conducted follow-up at a single time point of only three months. Finally, follow-up in our study was conducted via telephone interview or mailed questionnaire instead of a clinical visit, which may increase the risk of reporting bias.

## Conclusion

The present study provides clear evidence that IC occurred in more than one thirds of LHI patients at stroke onset. Independent risk factors were initial stroke severity assessed by baseline NIHSS score and atrial fibrillation, while dyslipidemia appeared to protect against IC at stroke onset. IC at stroke onset was associated with a higher frequency of stroke-related complications, especially brain edema and pneumonia. LHI patients with IC at stroke onset had higher rates of in-hospital death, 3-month mortality, and 3-month unfavorable outcome. And for this reason, IC at stroke onset should be considered a meaningful sign in LHI patients when developing treatment and management strategies, especially for patients involving non-dominant hemispheres.

## Supplementary information

Supplementary Information.

## Data Availability

The data that support the findings of this study are available from the corresponding author on reasonable request.
