# Supplementary material for: Impaired consciousness at stroke onset in large hemisphere infarction: incidence, risk factors and outcome
Source: Sci Rep. 2020 Aug 5;10:13170. doi: 10.1038/s41598-020-70172-1 (PMC7406648; doi:10.1038/s41598-020-70172-1)
Supplement: Supplementary file 1 — Supplementary Information. [file 41598_2020_70172_MOESM1_ESM.docx]

**Impaired consciousness at stroke onset in large hemisphere infarction: incidence, risk factors and outcome**

Jie Li**^a, b^**, MD; Ping Zhang**^b^**, MD; Simiao Wu **^a^**, PhD; Ruozhen Yuan**^a^**, MD; Junfeng Liu^a^, PhD; Wendan Tao^a^, PhD; Deren Wang^a^, PhD; Ming Liu^a^, MD, PhD

**^a^ Center of Cerebrovascular Diseases, Department of Neurology, West China Hospital, Sichuan University, Chengdu, PR China.**

**^b^ Department of Neurology, People’s Hospital of Deyang City, Deyang, PR China.**

***Co-corresponding author:**

**Ming Liu, MD, PhD, Center of Cerebrovascular Diseases, Department of Neurology, West China Hospital, Sichuan University, No. 37 GuoXue Xiang, Chengdu, Sichuan Province 610041, PR China. E-mail:** [wyplmh@hotmail.com](mailto:wyplmh@hotmail.com)**.**

**Jie Li, MD, Center of Cerebrovascular Diseases, Department of Neurology, West China Hospital, Sichuan University, No. 37 GuoXue Xiang, Chengdu, Sichuan Province 610041, PR China; Department of Neurology, People’s Hospital of Deyang City, No. 173, North Taishan Road, Deyang, Sichuan Province 618000, PR China. E-mail:** lijie860114@163.com.

**Supplemental table 1. Association between AF and impaired consciousness at stroke onset according to different characteristics.**

| **Variables** | **No. of patients** | | **No. of events (%)** | | ***Adjusted OR (95%CI)** | ***P* for interaction** |
| --- | --- | --- | --- | --- | --- | --- |
|  | **AF** | **Non-AF** | **AF** | **Non-AF** |  |  |
| **Stroke severity** |  |  |  |  |  | 0.52 |
| NIHSS <15 | 51 | 83 | 10(19.6) | 13(15.7) | 1.62 (0.15-17.10) |  |
| NIHSS≥15 | 60 | 62 | 42(70.0) | 28(45.2) | 4.34 (0.79-23.72) |  |
| **hyperlipidemia** |  |  |  |  |  | 0.24 |
| Yes | 19 | 28 | 6(31.6) | 4(14.3) | inf. (0.00-Inf.) |  |
| No | 92 | 117 | 46(50.0) | 37(31.6) | 2.59 (0.62-10.81) |  |

*** Each stratification adjusted for age, previous ischemic stroke, stroke in dominant hemisphere, TOAST classification, except for the stratification factor itself.**

**Abbreviations: AF= atrial fibrillation; NIHSS= National Institutes of Health Stroke Scale.**

**Supplemental table 2. Association between dyslipidemia and impaired consciousness at stroke onset according to different characteristics.**

| **Variables** | **No. of patients** | | **No. of events (%)** | | ***Adjusted OR (95%CI)** | ***P* for interaction** |
| --- | --- | --- | --- | --- | --- | --- |
|  | **Dyslip** | **Normal-lip** | **Dyslip** | **Normal-lip** |  |  |
| **Stroke severity** |  |  |  |  |  | 0.23 |
| NIHSS <15 | 28 | 106 | 4(14.3) | 19(17.9) | 0.71 (0.21-2.46) |  |
| NIHSS≥15 | 19 | 103 | 6(31.6) | 64(62.1) | 0.24 (0.08-0.79) |  |
| **AF** |  |  |  |  |  | 0.71 |
| Yes | 19 | 92 | 6(31.6) | 46(50) | 0.33 (0.09-1.20) |  |
| No | 28 | 117 | 4(14.3) | 37(31.6) | 0.46 (0.12-1.70) |  |

*** Each stratification adjusted for age, previous ischemic stroke, stroke in dominant hemisphere, TOAST classification, except for the stratification factor itself.**

**Abbreviations: Dyslip=dyslipidemia; Normal-lip=normal lipidemia; AF= atrial fibrillation; NIHSS= National Institutes of Health Stroke Scale.**

**Supplemental table 3. Outcome of the IC group involving dominant vs. non-dominant hemisphere.**

|  | **IC in DH**  **(N=56)** | **IC in NDH**  **(N=37)** | ***P value*** |
| --- | --- | --- | --- |
| Death in-hospital | 13(23.21) | 9(24.32) | 0.902 |
| 3-month case-fatality | 25(44.64) | 21(56.76) | 0.200 |
| 3-month unfavorable outcome | 32(57.14) | 28(75.68) | 0.043 |

**Abbreviations: IC= impaired consciousness at stroke onset; DH=** **dominant hemisphere; NDH=non-dominant hemisphere**
